# One health surveillance strategy for coronaviruses in Italian wildlife

**DOI:** 10.1017/S095026882300081X

**Published:** 2023-06-01

**Authors:** Stefania Leopardi, Rosanna Desiato, Matteo Mazzucato, Riccardo Orusa, Federica Obber, Daniela Averaimo, Shadia Berjaoui, Sabrina Canziani, Maria Teresa Capucchio, Raffaella Conti, Santina di Bella, Francesca Festa, Luisa Garofalo, Davide Lelli, Maria Paola Madrau, Maria Lucia Mandola, Ana Maria Moreno Martin, Simone Peletto, Silvia Pirani, Serena Robetto, Claudia Torresi, Maria Varotto, Carlo Citterio, Calogero Terregino

**Affiliations:** 1 Istituto Zooprofilattico Sperimentale delle Venezie, Legnaro, Italy; 2 Department of Veterinary Medicine, Università Aldo Moro di Bari, Valenzano, Italy; 3 Istituto Zooprofilattico Sperimentale del Piemonte, Liguria e Valle d’Aosta, Quart, Italy; 4 National Reference Center Wildlife Diseases, Aosta Valley, Quart, Italy; 5 Istituto Zooprofilattico Sperimentale di Abruzzo e Molise, Teramo, Italy; 6 Istituto Zooprofilattico Sperimentale della Lombardia ed Emilia Romagna, Brescia, Italy; 7 Department of Veterinary Sciences, Centro Animali Non Convenzionali (C.A.N.C), University of Turin, Turin, Italy; 8 Istituto Zooprofilattico Sperimentale di Lazio e Toscana, Roma, Italy; 9 Istituto Zooprofilattico Sperimentale della Sicilia, Palermo, Italy; 10 Molecular Medicine PhD Program, Department of Medicine and Surgery, University of Parma, Parma, Italy; 11 Istituto Zooprofilattico Sperimentale della Sardegna, Cagliari, Italy; 12 Istituto Zooprofilattico Sperimentale di Umbria e Marche, Perugia, Italy

**Keywords:** Coronaviruses, one health, surveillance, wildlife, zoonoses

## Abstract

The recent reinforcement of CoV surveillance in animals fuelled by the COVID-19 pandemic provided increasing evidence that mammals other than bats might hide further diversity and play critical roles in human infectious diseases. This work describes the results of a two-year survey carried out in Italy with the double objective of uncovering CoV diversity associated with wildlife and of excluding the establishment of a reservoir for SARS-CoV-2 in particularly susceptible or exposed species. The survey targeted hosts from five different orders and was harmonised across the country in terms of sample size, target tissues, and molecular test. Results showed the circulation of 8 CoV species in 13 hosts out of the 42 screened. Coronaviruses were either typical of the host species/genus or normally associated with their domestic counterpart. Two novel viruses likely belonging to a novel CoV genus were found in mustelids. All samples were negative for SARS-CoV-2, with minimum detectable prevalence ranging between 0.49% and 4.78% in the 13 species reaching our threshold sample size of 59 individuals. Considering that within-species transmission in white-tailed deer resulted in raising the prevalence from 5% to 81% within a few months, this result would exclude a sustained cycle after spillback in the tested species.

## Introduction

Zoonoses have been affecting humans since ancient times, involving the transmission of pathogens from domesticated animals and wildlife. Some of these, such as HIV/AIDS and COVID-19, adapted to people and are no longer transmitted from animals, leaving evidence of previous spillover events. While the burden of these human diseases is currently unrelated to their original source, it points out the challenge of managing pandemics once host transfer has occurred, and should encourage integrated research, surveillance, and capacity building within the framework of ‘One Health’. This approach is critical to improve our understanding of the ecology of pathogens within their natural hosts and the factors increasing risks for human health [1].

Coronaviruses (CoVs) are under the spotlight as important agents of human novel Emerging Infectious Diseases (nEID), which pose a great threat due to the lack of knowledge about their characteristics and control strategies and due to their increased pandemic potential caused by the fact that populations are often immunologically naïve [1]. Indeed, SARS-CoV, MERS-CoV, and SARS-CoV-2 have been responsible for severe epidemics after shifting from animal sources, peaking in the current pandemic of COVID-19. Strong evidence supports that also HCoV-HKU1, HCoV-OC43, HCoV-229E, and HCoV-NL63 originated from animals before adapting as human viruses causing mild flu-like symptoms [2, 3].

Among seven human CoVs, five share their evolutionary history with bat viruses, supporting the critical role of these animals for human nEID. Indeed, bats are by far associated with the highest diversity among known CoVs. Whether related to a particular relationship between bats and coronaviruses or more simply to the high biodiversity within the order Chiroptera, such a diversity strongly increases the likelihood that also future nEID might emerge from these animals. However, the evidence for direct transmission from bats to humans is still lacking, while the role of other domestic and wild mammals as a bridge or amplifying hosts is widely supported in all cases [3]. In addition, OC43 and HKU1 do not share any ancestry with bat viruses but are rather related respectively with variants found in livestock and rodents [2, 4]. This scenario highlights the need to revisit current surveillance activities for CoVs that, up to now, have largely been focused on bats. Monitoring should include the screening of other mammals, not only as a reaction to specific epidemics but also to gain a better knowledge of the diversity and distribution of CoVs. This approach would help to unravel evolutionary pathways and dynamics that might lead to the emergence of novel threats for humans [5–7].

In the aftermath of COVID-19, veterinary surveillance would also be critical to monitor SARS-CoV-2 as a zooanthroponosis, defined as an infection maintained by humans and naturally transmissible to animals [1]. During the pandemic, this virus infected a wide range of domestic, companion, wild, and laboratory mammals, caused outbreaks in European farmed minks (*Neovison vison*) [8], and, more recently, established a sustained infection cycle in white-tailed deer (*Odocoileus virginianus*) of North America [9]. Extensive genetics and phylogenetic studies showed that animal sequences are associated with multiple lineages that circulated in humans and that no crucial mutations were fixed during amplification events. This finding supports the assumption that SARS-CoV-2 was and still is readily able to infect animals, despite its significant adaptation to humans [10]. The infection of animals with SARS-CoV-2 may impact on both public and animal health, depending on its ability to cause disease or to transmit, amplify, and evolve in the new host [11]. Even if the current epidemiology of COVID-19 implies that people will be far more likely to acquire infections from other individuals, the establishment of an animal reservoir would increase challenges related to the control of the pandemic in the future and could sustain the emergence of novel variants showing increased pathogenicity, transmissibility, or resistance to prophylactic and therapeutic agents [12].

The present study reports the results from the surveillance program for CoVs circulating in wildlife implemented in Italy between 2020 and 2022. Within this framework, regional veterinary laboratories exploited existing programs of passive surveillance to collect and screen carcasses of different wild mammals with a harmonised approach, including the selection of target species and tissue and the molecular method used. The study highlighted the importance of strengthening and harmonising surveillance programs for pathogens along the human–wildlife interface, provided novel insights into the ecology of CoVs circulating in European wildlife, and excluded high circulation of SARS-CoV-2 within tested populations.

## Methods

### Screening

This survey was implemented and harmonised across Italy by eight laboratories belonging to the network of the Istituti Zooprofilattici Sperimentali, in collaboration with the University of Turin for sample collection. In order to maximise sample size and obtain robust data, we targeted specific hosts, including species that were known reservoirs of CoVs (i.e., bats), potential reservoirs of CoVs associated with the domestic counterpart (i.e., wild canids), considered particularly susceptible to SARS-CoV-2 (i.e., cervids), or particularly exposed to spillback events due to their urban and synanthropic habits (i.e., foxes) (Table 1). For all species, we aimed to test at least 59 individuals in order to secure a minimum detectable prevalence of 5% or lower with a 95% confidence. However, we also screened a few individuals or rarer species, whose results were analysed together with hosts from the same family/order. We obtained carcasses exploiting passive surveillance for animal diseases and in collaboration with wildlife rescue centres (WRC). During necropsies, we determined the sex, age, and physiological status of all animals and collected samples of lungs and intestines.

**Table 1.** Samples analysed in the study

Order	Family	Species	No. of testing partners	positive/sampled individuals	Observed prevalence (IC 95%)^a^	Minimum detectable prevalence (IC 97.5%)^b^
Artiodactyla	Bovidae (*Capra ibex, Ovis musimon, Rupicapra rupicapra*), Cervidae (*Capreolus capreolus, Cervus elaphus, D. dama*), Suidae (*S. scrofa*)		8	5/839	0.6 (0.19–1.39)	
	Bovidae		3	0/55	–	
	Cervidae		7	5/525	0.95 (0.31–2.21)	
		*C. capreolus*	6	5/354	1.41 (0.46–3.27)	0.83 (0–1.04)
		*C. elaphus*	3	0/103	–	2.85 (0–3.52)
		*Dama dama*	4	0/68	–	4.3 (0–5.28)
	Suidae	*Sus scrofa*	7	0/259	–	1.14 (0–1.41)
Carnivora	Canidae (*C. lupus, Vulpes vulpes*), Felidae (*Felis silvestris*), Mustelidae (*Lutra lutra, M. foina, Martes martes* − 1 positive/27 tested-, *Mustela nivalis*), Procyonidae (*Procyon lotor*), Ursidae (*Ursus arctos* − 1 positive/5 tested)		8	21/1374	1.53 (0.95–2.33)	
	Canidae		8	15/791	1.9 (1.07–3.11)	
		*Canis lupus*	4	7/199	3.52 (1.43–7.11)	1.48 (0–1.84)
		*V. vulpes*	8	8/592	1.35 (0.59–2.65)	0.49 (0–0.62)
	Mustelidae		6	5/566	0.88 (0.29–2.05)	
		*Martes foina*	6	0/182	–	1.62 (0–2.00)
		*Meles meles*	6	4/350	any CoV 1.14 (0.31–2.9) meleCoV/αCoV1 0.57 (0.07–2.4)	0.84 (0–1.05)
Chiroptera	Miniopteridae (*Miniopterus schreibersii*); Molossidae (*Tadarida teniotis*); Rhinolophidae (*Rhinolophus hipposideros*); Vespertilionidae (*Eptesicus serotinus, Hypsugo savii, Myotis crypticus* − 1 positive/2 tested-, *Myotis daubentonii; Myotis mystacinus, Nyctalus leisleri, Pipistrellus sp., P. kuhlii, Pipistrellus pipistrellus* − 3 positive /4 tested- *Pipistrellus pygmaeus*; *Plecotus auritus* − 1 positive/2 tested-, *Vespertilio murinus*); non identified bats −3 positive/56 tested-		8	16/516	3.10 (1.78–4.99)	
	Molossidae	*T. teniotis*	2	0/93	–	3.16 (0–3.89)
	Vespertilionidae		7	16/422	3.79 (2.18–6.08)	
		*H. savii*	5	5/146	3.42 (1.12–7.81)	2.02 (0–2.5)
		*Pipistrellus kuhlii*	4	3/191	any CoV 1.57 (0.33–4.52) Nc bat αcov 1.05 (0.13–3.73) MERS-like CoV 0.52 (0.01–2.88)	1.54 (0–1.91)
Eulipotyphla	Erinaceidae	*Erinaceus europaeus*	6	43/373	11.53 (8.47–15.21)	0.79 (0–0.98)
Lagomorpha	Leporidae (*Lepus europaeus; Sylvilagus floridanus*)		4	0/55	–	
Rodentia	Hystricidae (*H. cristata*); Myocastoridae (*Myocastor coypus*); Muridae (*Mus sp., Mus musculus*, *Rattus sp., Rattus rattus, Rattus norvegicus*); Sciuridae (*Marmota marmota, Sciurus vulgaris*); Ghiridae (*Glis glis*);		1	1/154	0.65 (0.02–3.56)	
	Hystricidae	*Hystrix cristata*	4	1/61	1.64 (0.04–8.8)	4.78 (0–8.7)
Total			9	87/3311		

a The observed prevalence has been calculated only in case of viral detection within the order, the family and for species tested at a minimum sample size of 59 individuals.

b The minimum detectable prevalence has been calculated for all the species reaching the minimum sample size of 59 individuals regardless of the detection of CoVs, and is intended as the exclusion of any CoV species circulating at higher prevalence values upon all negative samples. In particular, this value has been used to estimate the level of circulation for the pandemic virus SARS-CoV-2.

Depending on the laboratory, samples were homogenised in sterile PBS using the TissueLyser (Qiagen, Hilden, Germany) or the Omni Bead Ruptor (Omni International, Bedford, USA); nucleic acids were extracted using MagMAX Viral/Pathogen-II/Core on KingFisher Magnetic Particle Processors (Thermo Fisher Scientific, Waltham, USA), DSP Virus/Pathogen Mini Kit on QIAsymphony (Qiagen), Maxwell RSC viral total nucleic acid purification kit on Maxwell^®^ RSC48 (Promega, Madison, USA), or QIAAMP Viral RNA Mini Kit (Qiagen). Molecular screening was performed using a pan-coronavirus nested RT-PCR [6]. All amplicons were sequenced using Sanger and considered positive when providing clear sequences showing the highest match with CoVs using BLAST.

### Phylogenetic analyses

We aligned original sequences using the online tool Mafft with the G-INS-I setting [13] with reference CoVs, CoVs showing the highest BLAST identity and CoVs associated with target hosts, sampled in Italy and abroad. We then inferred a maximum likelihood (ML) phylogenetic tree using phyML (version 3.0) implemented in Seaview (Lyon, France), employing the LG + G4 substitution model, a heuristic SPR branch-swapping algorithm, and SH-like branch supports [14], and edited it using iTol [15]. We used MEGA.7 to determine pairwise, within and between clusters genetic distances for taxonomic attribution. We classified viruses within existing subgenera based on their nucleotide identity with reference strains, using 77.6% and 71.7% as thresholds for alpha- and beta-CoVs, respectively, as suggested elsewhere [16]. In addition, we considered sequences sharing more than 90% amino-acid identity as probable members of the same species [17].

### Data recording, spatial and statistical analyses

A dedicated webGIS application allowed partners to record samples as georeferenced terms using different base-maps and to associate sampling date, host species, age, sex, and laboratory results. We implemented the server-side of the application in JAVA, running on the Tomcat application server, and the client-side in HTML5, combining different JavaScript frameworks to manage the user interface and spatial engine. Layers were generated using standards Web Services published by GeoServer. At the end of the survey, we downloaded WebGIS data as Excel files for downstream statistics and to generate maps showing the distribution of samples using qGIS.

In case of CoV detection, we calculated the observed prevalence within orders, within families, and for the species with sample size of 59 individuals or higher. For these species, we also determined the minimum detectable prevalence, allowing us to define that the circulation of any CoV was below a certain level upon the negativity of all samples. This data was calculated regardless of the detection of animal CoVs within the sample, with the main objective of defining the sensitivity of the surveillance in excluding the circulation of SARS-CoV-2. I Observed and minimum detectable prevalence were calculated assuming a large population, a test sensitivity of 100%, and a confidence of 95% and 97.5%, respectively, using Stata statistical software v17.0 (Stata Corp., College Station, TX, USA) and Epitools Epidemiological Calculators (Ausvet).

For *Merbecovirus Hedgehog coronavirus 1* (EriCoV) and *Alphacoronavirus 1* (αCoV-1), which were most frequently found in our sample, we evaluated the association between prevalence and the qualitative variables sex, age, and tissue, using the Chi-square test implemented in the online tool WinEpi.

## Results

**Figure 1.**
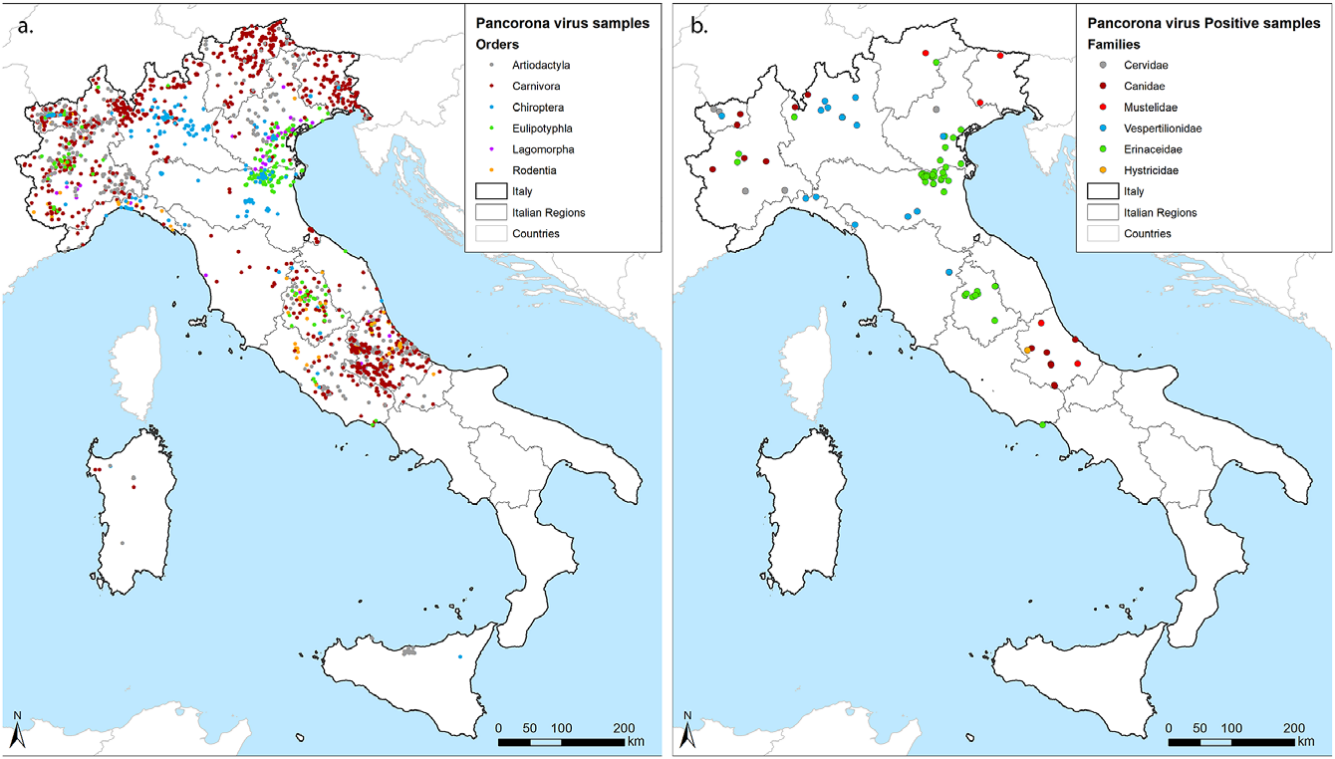
Geographical distribution of samples. (a) georeferenced identification of all samples included in the study coloured by the Order of hosts. (b) Positive samples, coloured by the family of hosts. We used grey for Cervidae within Artiodactyla, red for Canidae (Bordeaux) and Mustelidae (standard red) within Carnivora, blue for Vespertilionidae within Chiroptera, green for Erinacidae within Eulipotyphla and orange for Histricidae within Rodentia, as shown within figures.

Samples included in this survey were collected from 9 partners covering 1 to 3 Italian regions each, resulting in a good geographical coverage (Figure 1a). Although the number and type of species analysed differed among laboratories, animals of the orders Artiodactyla, Carnivora, and Chiroptera were sampled by 8 out of 9 partners, while the European hedgehog (*Erinaceus europaeus*), order Eulipotyphla, was targeted by 6. Four partners also provided partial data for rodents and lagomorphs. Thirteen of the target species reached the threshold of 59 individuals, with the red fox (*Vulpes vulpes*) obtaining the highest geographical coverage (Table 1). In total, 87 out of 3,311 screened individuals tested positive for CoVs in the lung (n:22), the intestine (n:62), or in both organs (n:9) across the territory (Figure 1b). Positive hosts belonged to 13 out of 42 tested animal species.

Hedgehogs accounted for almost half of the positive individuals (43/87) and showed the highest prevalence (11.53%) if compared to Chiroptera (3.1%), Carnivora (1.53%), Rodentia (0.65%), and Artiodactyla (0.6%) (Table 1). We obtained negative results across members of the Lagomorpha. By considering the sample size of 55, the prevalence of any CoV in this order can be considered lower than the minimum detectable prevalence of 5.3% (LC 95%). Focusing on the species level, we determined CoV prevalence in seven hosts other than hedgehogs, including the grey wolf (*Canis lupus*) (3.52%), the Savi’s pipistrelle (*Hypsugo savii*) (3.42%), the Kuhl’s pipistrelle (*Pipistrellus kuhlii*) (1.57%), the crested porcupine (*Hystrix cristata*) (1.64%), the roe deer (*Capreolus capreolus*) (1.41%), the red fox (1.35%), and the Eurasian badger (*Meles meles*) (1.14%). In addition, we detected CoVs regardless of the small sample size in the pine marten (*Martes martes*) (1/27), the brown bear (*Ursus arctos*) (1/5), the cryptic myotis (*Myotis crypticus*) (1/2), the brown long-eared bat (*Plecotus auritus*) (1/2), and the common pipistrelle (*Pipistrellus pipistrellus*) (3/4).

**Figure 2.**
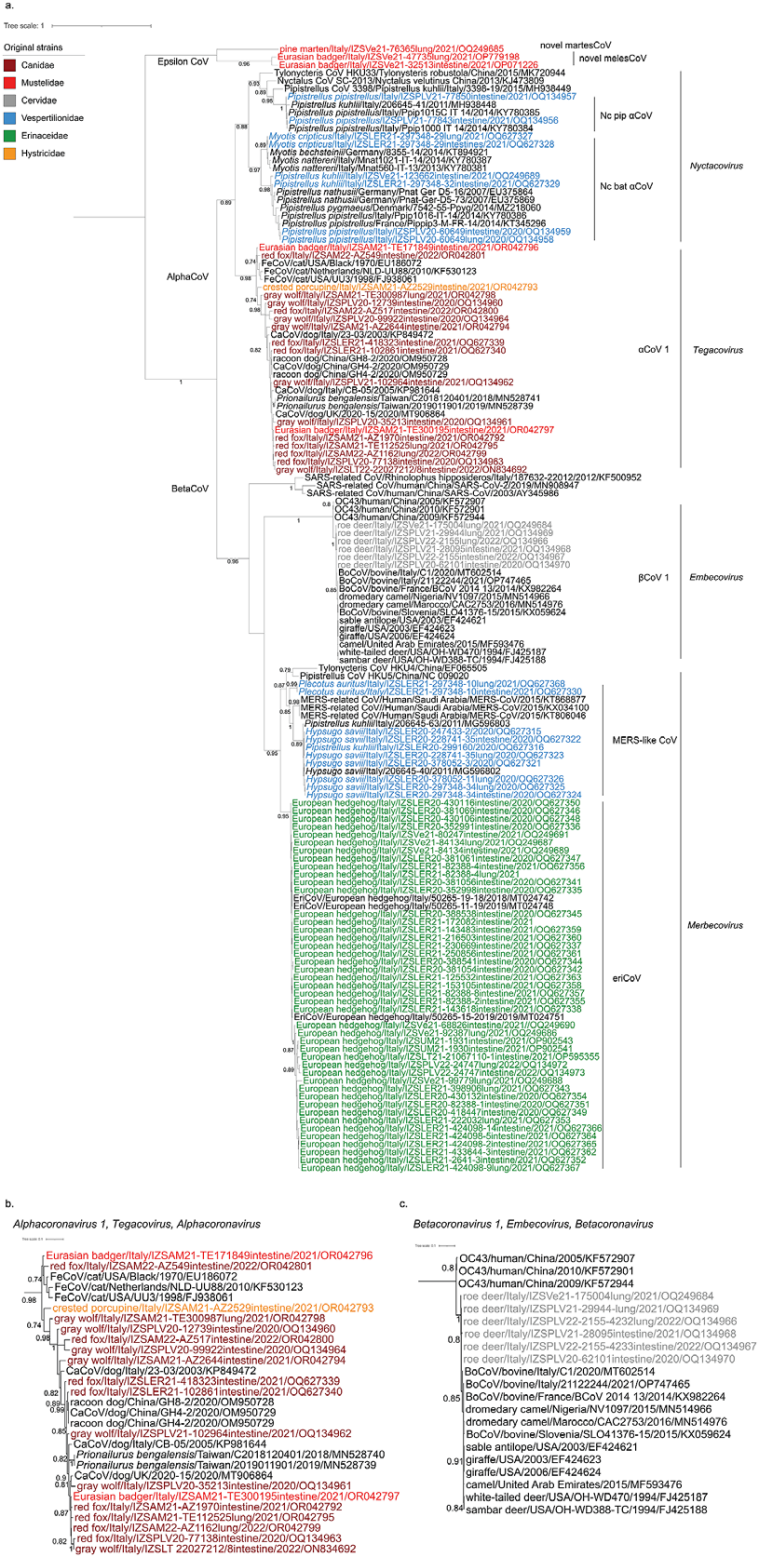
ML phylogenetic trees of coronaviruses found in the study. Original sequences from this survey are coloured based on the host family: we used the same colours of Figure 1, namely grey for Cervidae, bordeaux for Canidae, red for Mustelidae, blue for Vespertilionidae, green for Erinacidae and orange for Histricidae, as shown within the figure. (a) whole tree, (b) Pruned tree focused on Alphacoronavirus 1; (c) Pruned tree focused on Betacoronavirus 1.

**Figure 3.**
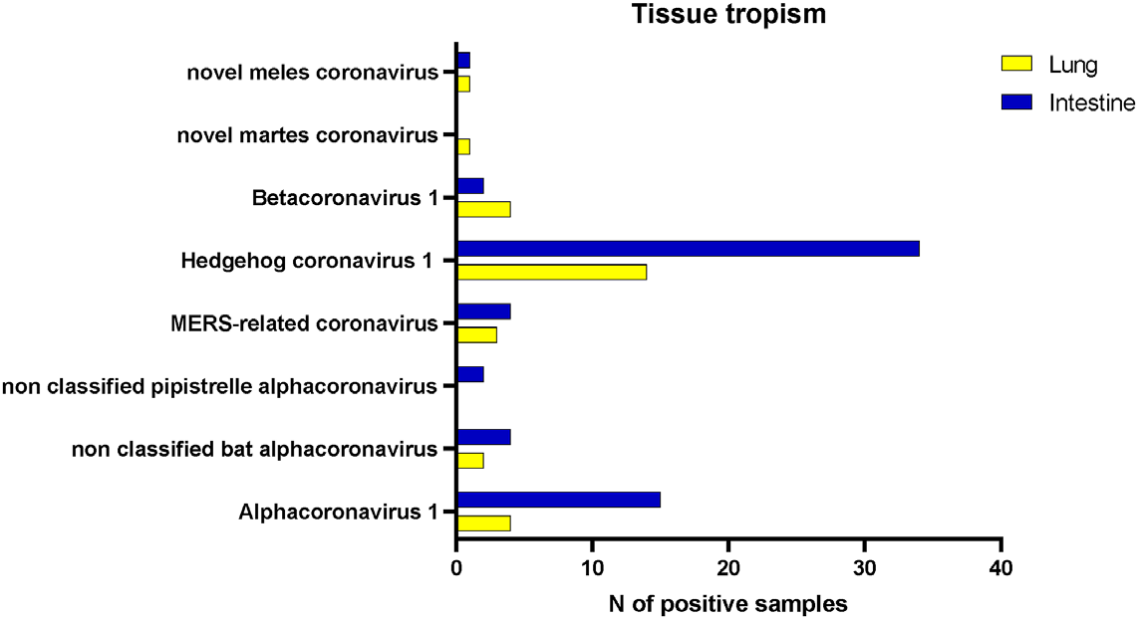
Positive results for lung and intestine.

An accurate classification of CoVs through whole genome sequencing (WGS) was beyond the objectives of this study. However, we were able to classify most strains within four subgenera (*Tegacovirus*, *Nyctacovirus*, *Embecovirus*, and *Merbecovirus*) based on the nucleotide identity in 440 base pairs of the *RNA-dependent RNA-polymerase* (*RdRp*). Our sequences fell within six supported clusters with amino-acid identity higher than 90% with known CoVs, suggesting their grouping within correspondent CoV species (Table 2, Figure 2a). We found two additional clusters in mustelids unrelated to any CoV, suggesting they belong to novel species hereafter referred to as meles coronavirus (melesCoV) and martes coronavirus (martesCoV). While most positive samples derived from the intestine, 7 out of 9 viruses were found in both the gastroenteric and the respiratory tracts (Figure 3).

**Table 2.** Host association of coronaviruses identified in the study

Genus	Alphacoronavirus			Betacoronavirus			Novel *Epsiloncoronavirus*		
Subgenus	Tegacovirus	Nyctacovirus		Merbecovirus		Embecovirus	novel	novel	
Putative species	αCoV 1	Nc pip αcov	Nc bat αcov	MERS-like cov	eriCoV	βCoV 1	novel melescov	novel martescov	neg
*Capreolus capreolus*	0	0	0	0	0	5	0	0	349
*C. lupus*	7	0	0	0	0	0	0	0	192
*Vulpes vulpes*	8	0	0	0	0	0	0	0	584
*Ursus arctos*	1	0	0	0	0	0	0	0	4
*H. cristata*	1	0	0	0	0	0	0	0	60
*E. europaeus*	0	0	0	0	43	0	0	0	330
*Hypsugo savii*	0	0	0	5	0	0	0	0	141
*P. kuhlii*	0	0	2	1	0	0	0	0	188
*Pipistrellus pipistrellus*	0	2	1	0	0	0	0	0	1
*P. auritus*	0	0	0	1	0	0	0	0	1
*M. crypticus*	0	0	1	0	0	0	0	0	1
*M. martes*	0	0	0	0	0	0	0	1	26
*Meles meles*	2	0	0	0	0	0	2	0	346
Total individuals	19	2	5	7	43	5	2	1	

Scientific names of species and viruses have been abbreviated for graphical purposes. Please refer to the text for disclosure.

Most positive hosts were associated with CoVs that had already been described in the same species. Viruses of hedgehogs showed amino-acid identity of 96.5% with EriCoV, which is broadly described in the country (Table 2). The virus was predominantly found in the intestine (p-value:0.006; prevalence ratio:2.27) (Figure 3), but not associated with either sex (p-value:0.71) or age (p-value:0.89). Phylogenetic analyses showed different clusters of eriCoV but no evidence of geographical differentiation (Figure 2a). Similarly, bat CoVs found in the study were genetically related to alpha and betacoronaviruses found in the same genera elsewhere, confirming their host-specificity. The virus from *Myotis crypticus* was most closely related with strains found in *Myotis bechsteinii* from Germany and *M. nattereri* from Italy, while viruses from Kuhl’s and common pipistrelles were associated with Italian sequences from the same species (Figure 2a). Interestingly, viruses from myotis and pipistrelle bats formed two sister clades sharing 97.6% amino-acid identity, which suggests they belong to the same putative species, hereafter referred to as non-classified bat alpha-CoV (Nc bat-αCoV). Nc bat-αCoVs shared 77% nucleotide identity with either *Nyctalus velutinus alphacoronavirus SC2013* or *Alphacoronavirus HKU33,* allowing their putative classification within the subgenus *Nyctacovirus.* Two additional viruses from the common pipistrelle fell within another cluster sharing 86% amino-acid identity with Nc bat-αCoV and 83% nucleotide identity with the *Italian P. kuhlii coronavirus 3398*, suggesting it likely defines a separate species within the subgenus *Nyctacovirus*, hereafter referred to as non-classified pipistrelle alphacov (Nc pip-αCoV). Phylogenetic and genetic analyses showed that all other bat sequences belong to the species *MERS-related coronavirus,* subgenus *Merbecovirus* (85% and 97% mean nucleotide and amino-acid identity). As for Nc bat-αCoV, bat MERS-like CoVs clustered upon the host, with one group including sequences from a brown long-eared bat, sampled here for the first time, and the other one shared between viruses from the genera *Hypsugo* and *Pipistrellus.* Both clusters were related to but distinct from MERS-CoV (Figure 2a).

As expected, we confirmed that wild mammals may be infected with viruses associated with their domestic counterpart. Among these, we found *Bovine coronavirus* (BoCoV) (species *Betacoronavirus 1,* subgenus *Embecovirus*) in the roe deer (99.1% mean amino-acid identity) and αCoV-1 (subgenus *Tegacovirus*) in wild canids, the brown bear, the badger, and the crested porcupine (97.1% mean amino-acid identity) (Table 2). αCoV-1 showed gastroenteric tropism in wildlife as in pets (p-value:0.016; prevalence ratio:3.53), but it was also detected in the lungs of foxes and wolves (Figure 3). Despite sequencing of the whole genome being necessary for a correct taxonomic placement, partial sequences from this study fell into two phylogenetic groups related with *Feline coronavirus* (FeCoV) or *Canine coronavirus* (CaCoV) but showed no clustering upon the host species (Figure 2b). On the other hand, all sequences of BoCoV found in the roe deer clustered together in a separate clade compared to strains associated with cows in Italy and other ungulates sequenced elsewhere, with the main genetic distance of 2% at the nucleotide and 1% at the amino-acid level (Figure 2c).

Finally, we succeeded in the discovery of novel CoVs in mustelids. Among these, melesCoV have been characterised through next-generation sequencing as belonging to a new putative *Epsiloncoronavirus* genus within the family *Coronaviridae* [18] (Zamperin et al., 2023, under review). The virus was found in the intestine and lungs of two individuals, with amino-acid identity of 92.4% supporting their placement under the same species. On the other hand, no further sequencing was possible for martesCoV, found in the lung of a pine marten, due to the low quality of the sample. Based on partial *RdRp,* the sequence clustered with melesCoV within the new genus, but a low amino-acid identity of 63.2% supports its classification as a distinct species in a different subgenus.

As a major result, all samples were negative for SARS-CoV-2. Depending on the sample size reached among the consortium, the minimum detectable prevalence ranged between 0.49% and 4.78% (mean:1.99%), thus excluding the presence of the virus at higher levels in the tested species (Table 1).

## Discussion

This study presents the results of passive surveillance for CoVs implemented in Italian wildlife during the pandemic of COVID-19, similar to other countries [19–23]. By standardising the sampling approach to orders particularly susceptible or exposed to CoVs, the survey aimed at enhancing our knowledge of the host range of these viruses that, up to now, have been biased by different surveillance efforts in different animal groups. Indeed, wide-spectrum molecular approaches for the identification of novel CoVs have been historically implemented in bats, while domestic mammals and other wildlife were mostly screened in response to specific emergencies targeting specific CoV species [5, 6].

Due to the opportunistic nature of passive surveillance, we were not always able to reach the aimed sample size, but succeeded in screening at least 59 individuals for 13 species from five orders. Our data confirmed that, even in the absence of sampling biases, bats retain the highest number of CoVs across orders, likely due to their ancient evolutionary time and consequent diversification, providing various receptors for viruses and ways of exposure through different diets and ecological niches [7, 24]. Bats showed the highest CoV diversity also at the genus/species level, with *P. kuhlii* and *H. savii* being infected with two putative CoV species described in these same animals across Europe, including Italy [25–27]. This finding suggests that the diversification of bat CoVs might also be driven by more frequent recombination events favoured by multiple infections [28].

Interestingly, also the Eurasian badger was infected with two CoVs, namely the novel melesCov and αCoV-1. Despite this latter finding supporting previous serological data [29], the two sequences of αCoV-1 from Italian badgers sampled in the same area were not related to each other, while they clustered with CaCoV and FeCoV from domestic and wild animals, suggesting they might result from cross-species transmissions. In addition, we cannot exclude the finding as related with the infection of prey contaminating the intestinal tract of the tested animals. Regardless of the role of badgers in the ecology of αCoV-1, our results point out that mustelids are another group that deserves particular attention. The affinity with diverse coronaviruses has already been supported by their susceptibility to viruses from three different genera, including the *Gammacoronavirus* found in Chinese ferret-badgers (*Melogale moschata*) [30], three *Alphacoronavirus* of the subgenus *Minacovirus*, and the *Betacoronavirus* SARS-CoV-2, that are pathogenic for domestic ferrets (*Mustela putorius furo*) and minks (*N. vison*) [8, 29, 31]. Our discovery of two novel CoVs within the family adds up perfectly to this evidence and suggests they might still hide a wide viral diversity.

We detected no infections in rodents, besides the occasional finding of αCoV-1 in porcupines. This result is in contrast with recent evidence obtained worldwide that supports such a high frequency and diversity of CoVs in this vast animal group that could mirror what has been uncovered in bats in the past 20 years [4]. However, rodents have been screened only marginally in this survey and the threshold sample size was only achieved for porcupines (61 tested individuals, minimum detectable prevalence:4.78%). Because our results support that the observed prevalence of CoVs in wild animals screened through passive surveillance ranges between 1% and 3%, we suggest that opportunistic testing might not be effective for CoV detection. In this context, our negative findings in rodents and other species investigated with low coverage might be related to the small sample size other than the absence of infection. In line with what has been suggested by Anthony and colleagues, we suggest that a sample size of around 300 individuals per species would be more effective to exclude the association of animals with CoVs, by detecting a prevalence as low as 1% [18].

Compared to our average data, hedgehogs sustained an extraordinary prevalence of 11.53%, within the range between 10% and 50% previously described in Europe and Asia [32, 33]. However, considering that our sample included several individuals that had died in WRCs, our data might partially be confounded by amplification of the infection during captivity, as suggested elsewhere [34]. In bats, whose samples are also frequently submitted to laboratories by WRCs, we found a 3.1% percentage of positivity, with prevalence ranging between species from 0 to 3.42%. This data is consistent with previous reports analysing carcasses [34] and suggests a lower amplification of bat CoVs during captivity, which could be associated with more frequent individual housing, lower animal density within group cages, or shorter survival after admission that prevents extensive transmission events. While supporting their critical role in the evolution of coronaviruses, our data showed low prevalence in mustelids across (0.88%) and within species (0.57% for melesCoV in the badger), in line with the positivity rate of 1.1% recorded for the *Gammacoronavirus* of Chinese ferret-badgers [30]. This evidence is likely associated with the solitary habits of mustelids and could explain the low detection rate in surveillance programs and the low implication in spillover events [8].

Other than uncovering the diversity of CoVs associated with wild animals, we described their circulation at the wild-domestic interface. Our finding of CaCoV, FeCoV, and BoCoV in several species confirmed their broad host range, as previously suggested [35–38]. Prevalence data of αCoV-1 in foxes and wolves mirrored average results from other species, suggesting either maintenance of the virus or a high frequency of spillovers from pets. If on one hand the fact that our CaCoV and FeCoV sequences were interleaved with pet strains would not support the existence of a feral cycle, on the other we cannot exclude limited transmission within single species or in a multi-species system, as suggested for the Serengeti ecosystem [36]. While a more targeted surveillance plan paired with WGS could clarify the ecology of *Alphacoronavirus 1*, the susceptibility of several carnivores poses a conservation concern in case of clinical disease, that would be most likely related with the highly pathogenic *feline infectious peritonitis virus* [35]. Fortunately, positive individuals from this study were not associated with relevant clinical signs or lesions, similar to what was described for pets and in other wildlife surveys [36]. On the other hand, all BoCoVs sequenced from the roe deer formed a sister clade to viruses associated with local bovines, suggesting these animals might maintain their own viral strain. In this context, WGS could clarify whether these viruses underwent a process of host adaptation, as determined for giraffe’s BoCoVs [38, 39].

The molecular method used in this study has a high sensitivity towards the pandemic SARS-CoV-2, so that the lack of detection excludes its high circulation in tested animals, among which mustelids, cervids, and synanthropic species should be considered at highest risk of becoming a wildlife reservoir. Up to date, relevant within-species transmission has followed spillback of SARS-CoV-2 from humans to wild animals only in the case of the white-tailed deer, with the first bell rung by serological studies in 2020 [20]. Subsequent molecular investigations confirmed the infection with multiple human lineages, and within-species amplification was supported by phylogenetic evidence and by the sharp increase in prevalence from 5.1% in September to 81.3% in December 2020 [40]. As noted, the sensitivity of our screening varied depending on the sample size obtained for each species, with the minimum detectable prevalence as low as 0.84% in the badger, which is widespread in Europe and characterised by higher sociability compared to other mustelids, 0.83% in the roe deer, the European species more closely related to the white-tailed deer, and 0.49% in the synanthropic red fox.

## Conclusion

Besides the specific results obtained, our survey identified several parameters that could be used in building, strengthening, or harmonising veterinary capacities in the framework of ‘One Health’, particularly to enhance our knowledge on coronaviruses infecting wildlife that could be transmitted to humans in the future, and to manage the risks related to the possible spillback of SARS-CoV-2 from people to wildlife.

Our study underlines that, in order to fulfil these objectives, surveillance should target selected species to be sampled with sufficient coverage. While the epidemiology of SARS-CoV-2 may vary in different hosts depending on their susceptibility, population density, and social behaviour, we determined in 59 individuals the minimum sample size for detection of animal outbreaks, excluding 5% prevalence that characterised the initial amplification of SARS-CoV-2 in white-tailed deer. While the detection of all spillback cases would be ineffective, our data suggest that increasing the sample size to 300 samples would exclude 1% circulation of CoVs, allowing more accurate studies of the ecology and epidemiology of CoVs and early detection of SARS-CoV-2.

Target species should be determined based on updated knowledge of CoVs, including animals’ susceptibility to SARS-CoV-2. Currently, we suggest rodents and mustelids as a priority target to further unveil the diversity of the family *Coronaviridae,* and hedgehogs as hosts for *Merbecoviruses* whose zoonotic potential is yet to be determined. In addition, bats are still a relevant target due to their association with a wide variety of CoVs and their frequent implication as a source for human nEID. However, to provide novel insights, bat surveillance should be focused on specific species, each one sampled with a good sample size and correct identification. Cervids, mustelids, and bats of the genus *Rhinolophus* are still considered at highest risk for the spillback of SARS-CoV-2. In addition, synanthropic rodents, carnivores, and artiodactyls have the highest likelihood of exposure and should not be overlooked. Because spillbacks also bare risks for conservation and animal health, syndromic surveillance should be implemented to exclude SARS-CoV-2 from mortality events in any wild species.

While we corroborated previous evidence that animal CoVs are mostly found in the intestinal tract, we have succeeded in detecting most viruses also in the lungs, the main target of SARS-CoV-2. Thus, we suggest testing of both tissue as the most effective approach. Finally, the depth of molecular data generated from surveillance should depend on the specific objectives of each plan. While WGS should be the preferred approach whenever possible, partial sequences of *RdRp* generated in this study provided sufficient information for a preliminary assessment of diversity, prevalence, and distribution data.

Overall, we believe that the unbiased pan-coronavirus surveillance implemented in Italy reached a good balance between costs and expected outcomes, which allowed us to detect known and unknown CoVs in target animals and to exclude the amplification of SARS-CoV-2 in the tested populations.

## Data Availability

WebGIS data generated from this study are stored with the PostGIS extension on a PostGreSQL database. Partial *RdRp* sequences have been deposited in Genbank under accession numbers ON834692, OP595355, OP902541, OP902543, OQ134956-OQ134964, OQ134966-OQ134970, OQ134972, OQ134973, OQ249684-OQ249692, OQ627315-OQ627316, OQ627321- OQ627330, OQ627335- OQ627368, and OR042792-OR042801.
