# One health surveillance strategy for coronaviruses in Italian wildlife – CORRIGENDUM

**DOI:** 10.1017/S0950268823001000

**Published:** 2023-06-27

**Authors:** Stefania Leopardi, Rosanna Desiato, Matteo Mazzucato, Riccardo Orusa, Federica Obber, Daniela Averaimo, Shadia Berjaoui, Sabrina Canziani, Maria Teresa Capucchio, Raffaella Conti, Santina di Bella, Francesca Festa, Luisa Garofalo, Davide Lelli, Maria Paola Madrau, Maria Lucia Mandola, Ana Maria Moreno Martin, Simone Peletto, Silvia Pirani, Serena Robetto, Claudia Torresi, Maria Varotto, Carlo Citterio, Calogero Terregino

**Affiliations:** 1 Istituto Zooprofilattico Sperimentale delle Venezie, Legnaro, Italy; 2 Department of Veterinary Medicine, Università Aldo Moro di Bari, Valenzano, Italy; 3 Istituto Zooprofilattico Sperimentale del Piemonte, Liguria e Valle d’Aosta, Quart, Italy; 4 National Reference Center Wildlife Diseases, Aosta Valley, Quart, Italy; 5 Istituto Zooprofilattico Sperimentale di Abruzzo e Molise, Teramo, Italy; 6 Istituto Zooprofilattico Sperimentale della Lombardia ed Emilia Romagna, Brescia, Italy; 7 Department of Veterinary Sciences, Centro Animali Non Convenzionali (C.A.N.C), University of Turin, Turin, Italy; 8 Istituto Zooprofilattico Sperimentale di Lazio e Toscana, Roma, Italy; 9 Istituto Zooprofilattico Sperimentale della Sicilia, Palermo, Italy; 10 Molecular Medicine PhD Program, Department of Medicine and Surgery, University of Parma, Parma, Italy; 11 Istituto Zooprofilattico Sperimentale della Sardegna, Cagliari, Italy; 12 Istituto Zooprofilattico Sperimentale di Umbria e Marche, Perugia, Italy

When this article was originally published in Epidemiology & Infection, the funding statement was inaccurate. This has been updated in the article to the following:

Financial support. The study was supported by the Italian Ministry of Health through the Strategic Project ‘Ricerca Corrente IZSVE 01/20 RCS’. This research was partially supported by EU funding within the NextGenerationEU-MUR PNRR Extended Partnership initiative on Emerging Infectious Diseases (Project no. PE00000007, INF-ACT). Istituto Zooprofilattico Sperimentale dell’Abruzzo e del Molise (IZSAM) was also funded by the European Union’s Horizon 2020 Research and Innovation programme (One Health European Joint Programme under grant agreement No. 773830).
